# A Leaky Membrane and a Sodium Transporter at Life’s Great Divergence

**DOI:** 10.1371/journal.pbio.1001927

**Published:** 2014-08-12

**Authors:** Richard Robinson

**Affiliations:** Freelance Science Writer, Sherborn, Massachusetts, United States of America

The deepest branch on the evolutionary tree is between bacteria and archaea (eukaryotes arrived late, probably as a fusion of the two). These two domains of life share fundamental aspects of their biochemistries, including a genetic code and their reliance on cross membrane proton gradients to drive adenosine triphosphate (ATP) synthesis via the ATP synthase enzyme. Yet, the membranes themselves are profoundly different. In bacteria, glycerol phosphate links to an unbranched fatty acid via an ester bond (forming the same phospholipid structure found in all eukaryotes), while in archaea it links to a (typically) branched isoprenoid via an ether bond. The stereochemistry of the resulting molecules also differs, all of which strongly suggests that these membrane components evolved after the two diverged from the last universal common ancestor (LUCA).

As logical as it is, this conclusion has met with much head-scratching among evolutionary biologists, since it suggests that LUCA itself did not possess a modern membrane. However, if that is the case, how did it harvest energy, and what could drive its archaeal and bacterial progeny to diverge in the structure of their membrane lipids?

In this issue of *PLOS Biology*, Victor Sojo, Andrew Pomiankowski, and Nick Lane develop a mathematical model of bioenergetics in a LUCA-like cell, with a membrane lipid that could be a precursor of both types of modern membranes. Leakiness is a critical feature of this membrane, and the authors offer a plausible scheme for its evolution into the two distinct membranes found in bacteria and archaea today.

In modern cells, the proton gradients that drive ATP synthesis are generated by proton pumps in the membrane. However, like the membranes themselves, these pumps differ in archaea and bacteria. One possibility is that LUCA could have used proton gradients but not generated them itself and therefore might have relied on natural proton gradients. However, that leads straight to another problem. The influx of protons down a natural gradient can’t go on forever, or even very long, since the buildup of positive charge halts the electrostatic drive behind the process—hence, the authors reasoned, the value of a leaky membrane. Entering protons could either leak out or be neutralized by hydroxide ions leaking in, thereby maintaining the gradient and the ability to make ATP. Fatty acids without their glycerol phosphate head groups form just such a leaky membrane, since protons attaching to the weak negative charge on the fatty acid head neutralize it, allowing it to randomly flip back and forth within the membrane, carrying the proton out with it.

As a source of proton gradients, LUCA most likely relied on naturally occurring pH differences like those found in the oceans, where alkaline fluids seep from deep sea vents into relatively acidic seawater. The model they built then posited a cell in contact with a constant flow of protons on one side (from seawater), a constant flow of alkaline fluid on the other (potentially from a vent), and a leaky membrane containing an ATP synthase. They found that with a 3-unit pH gradient (i.e., a 1000-fold concentration gradient of protons) and the ATP synthase comprising 1% of the membrane, the cell could drive synthesis of ATP.

Movement away from deep sea vents into environments without natural proton gradients would have been impossible for this type of leaky cell, but the authors suggest that a second membrane protein—a sodium-proton antiporter (SPAP)—could have laid the first steps. SPAP exchanges a positively charged proton on one side of the membrane for a positively charged sodium on the other. Sodium doesn’t neutralize the fatty acid in the same manner and consequently leaks through much less. The continuous flux of protons through SPAP could therefore add a sodium gradient to the natural proton gradient, giving cells more power and therefore allowing them to survive on smaller proton gradients, facilitating early spread and divergence. SPAP is found in both archaea and bacteria, and the authors suggest it arose in LUCA.

Modern cells don’t just exploit proton gradients; they actively create them as well, and here, the existence of SPAP emerges as a crucial preadaptation for the evolution of the modern cell. In the absence of SPAP, pumping protons across a leaky membrane gives no benefit, as most of the protons immediately leak back through the membrane. Making the membrane less leaky doesn’t help, as that cuts off cells from the natural proton gradient, undermining their power. However, pumping in the presence of SPAP does pay. For every proton pumped out, a little extra energy is retained in a sodium gradient. That makes pumping with SPAP beneficial even with a leaky membrane. The authors showed that it reaped even bigger rewards as membranes tightened, giving for the first time an advantage to modern phospholipid membranes.

LUCA’s membrane may have contained both fatty acids and isoprenoids, and tightening is the natural consequence of adding a glycerol phosphate head to either membrane lipid. Based on their model, the authors suggest that the stereochemical differences between archaea and bacteria arose randomly in separate populations (which diverged with the benefit of SPAP). The authors point out that both the archaeal version and the bacterial version of glycerol phosphate arise from the same precursor (dihydroxyacetone phosphate). The two opposing stereochemistries arise from attacking the central atom from two opposing sides, likely a random event initially but one that eventually became fixed in separate populations.

Whether or not this model is correct in detail, it offers a robust and potentially testable hypothesis that goes a long way in explaining why archaea and bacteria are so alike and yet so different.

**Sojo V, Pomiankowski A, Lane N (2014) A Bioenergetic Basis for Membrane Divergence in Archaea and Bacteria.** doi:10.1371/journal.pbio.1001926

**Figure 1:**
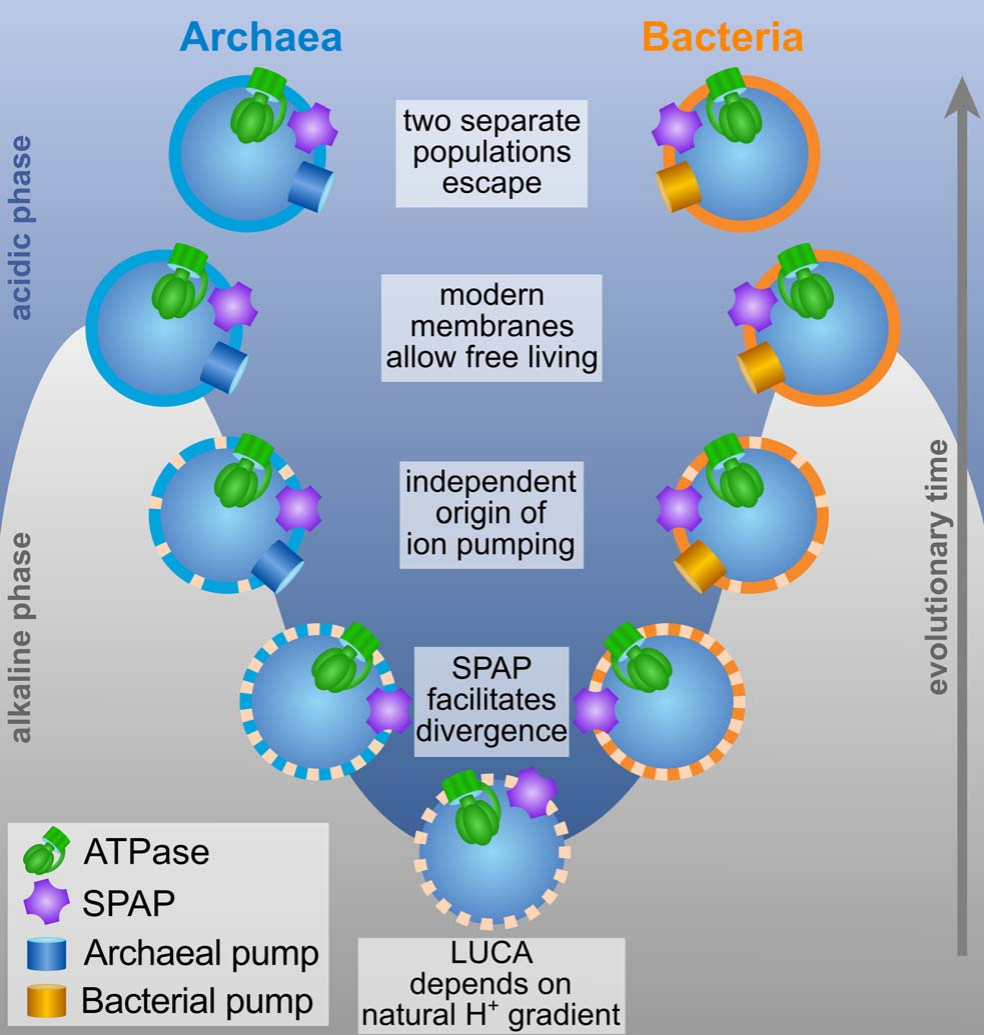
Why do bacteria and archaea differ so fundamentally in their membranes and other traits? It makes more sense if they started out living on natural proton gradients.

